# GNRB® Knee Arthrometer: Inter- and Intra-observer Reliability and Learning Curve

**DOI:** 10.7759/cureus.70838

**Published:** 2024-10-04

**Authors:** Pauline Unal, Ramy Samargandi, Antoine Schmitt, Hoel Letissier, Julien Berhouet

**Affiliations:** 1 Orthopedic Surgery, Centre Hospitalier Régional Universitaire (CHRU) de Tours, Tours, FRA; 2 Orthopedic Surgery, Centre Hospitalière d'Amboise, Amboise, FRA; 3 Orthopedic Surgery, Faculty of Medicine, University of Jeddah, Jeddah, SAU; 4 Orthopedic Surgery, Centre Hospitalier Universitaire (CHU) de Brest, Brest, FRA

**Keywords:** acl rupture, anterior cruciate ligament, anterior drawer test, gnrb arthrometer, knee, lachman test, laximeter

## Abstract

Background

Diagnosing anterior cruciate ligament rupture is challenging, particularly due to the subjective nature of clinical laxity assessments. Objective evaluation methods are necessary for consistency and publication in clinical research. This study aims to assess the reproducibility of the GNRB® knee arthrometer (GeNouRoB, Laval, France) across different examiners and to examine the associated learning curve for a junior examiner.

Methods

Anterior translation measurements were conducted on 20 healthy knees using the GNRB arthrometer. Two examiners, a senior and a junior, performed the measurements independently and were blinded to each other's results. Measurements were taken at two different push forces (134 N and 200 N). The study evaluated inter- and intra-observer reproducibility using Cohen's kappa coefficient and the intraclass correlation coefficient (ICC). The junior examiner also performed a third series of measurements to assess the learning curve.

Results

The senior examiner demonstrated excellent reproducibility with an ICC greater than 0.75 for all measurements. The junior examiner's reproducibility varied from fair to good, with an ICC ranging from 0.45 to 0.75. Inter-observer reproducibility between the senior and junior examiners was excellent (ICC >0.75). Notably, the junior examiner's reproducibility improved to an excellent level (ICC >0.75) during the second series of measurements.

Conclusion

The GNRB system provides a reproducible method for evaluating anterior knee laxity across different examiners. A learning curve of approximately 20 knees is sufficient for a junior examiner to achieve statistically excellent reproducibility.

## Introduction

Anterior cruciate ligament (ACL) ruptures are common knee injuries affecting athletes. The ACL is crucial for maintaining knee stability and preventing excessive translation of the tibia relative to the femur. ACL ruptures typically occur due to non-contact pivoting injuries such as sudden deceleration, landing from a jump, or abrupt changes in direction. Although less common, contact with direct blows or collisions can also cause ACL injuries [1,2]. An accurate diagnosis of an ACL rupture requires a detailed injury history, physical examination, and diagnostic imaging. In cases of ACL ruptures, most patients recount a history of hearing and feeling a sudden "pop" followed by immediate knee swelling, pain, and impaired knee stability. This greatly impairs their ability to perform physical activities. The clinical interrogation often looks for signs of subjective instability, and the physical examination looks for objective instability with the Lachman’s test, an anterior drawer test, and a pivot shift [3,4]. This instability is often obvious on clinical examination in case of a complete rupture. However, measurement is more subtle in the case of partial rupture or in the case of medium- or long-term postoperative analysis. Magnetic resonance imaging (MRI) allows confirmation of the diagnosis in addition to the search for associated meniscal lesions [2]. The diagnosis is often straightforward, but follow-up may require precise measurement of anterior laxity, particularly for the purpose of objective and examiner-independent measurements for publication purposes.

There are different measuring systems available: Telos® system with radiographic measurement (telos Arzt- und Krankenhausbedarf GmbH, Wölfersheim, Germany), KT 1000^TM^ and KT 2000^TM ^(MEDmetric® Corporation, San Diego, California, United States), and GNRB® (GeNouRoB, Laval, France). The advancements in clinical measurement assessment techniques have contributed to enhanced accuracy and objectivity in diagnosing and quantifying ACL injuries. However, challenges remain, including the variability in measurement outcomes due to factors such as patient positioning, examiner experience, and the choice of assessment method. Therefore, a comprehensive understanding of the strengths, limitations, and comparative reliability of these assessment techniques is crucial for clinicians, researchers, and healthcare providers involved in managing ACL tears.

The GNRB knee arthrometer has emerged as a promising instrument for accurately quantifying anterior tibial translation and evaluating ACL instability. It is designed to measure anterior tibial translation under controlled loads, offering a non-invasive means of assessing ACL integrity.

The primary objective of this study was to evaluate the intra- and inter-observer reproducibility of the GNRB system to define whether the system can allow objective and reproducible measurements of anterior knee laxity. The secondary objective was to evaluate the learning curve using this measurement system.

## Materials and methods

The study was conducted at the University Hospital of Tours (Hôpital Trousseau), Tours, France. The study protocol was approved by the Centre Hospitalier Régional Universitaire (CHRU) de Tours (approval number: N° 2020-024). All subjects provided informed consent.

Participants

We performed the measurements on a population of healthy knees from medical students who volunteered during their internship in the orthopedic department. We included five males and five females with no history of knee trauma. We carried out the measurements bilaterally to obtain 20 knees (10 right and 10 left).

Installation of the system and measurement

Two evaluators performed the measurements, a senior surgeon specializing in knee surgery and a junior resident wishing to specialize in sports medicine. The two assessors had never used the machine but had been trained by the GNRB supplier during two explanation sessions. These training sessions mainly concerned the installation of the patient, which strongly influences the measurement, and use of the software. Both evaluators received identical training.

**Figure 1:**
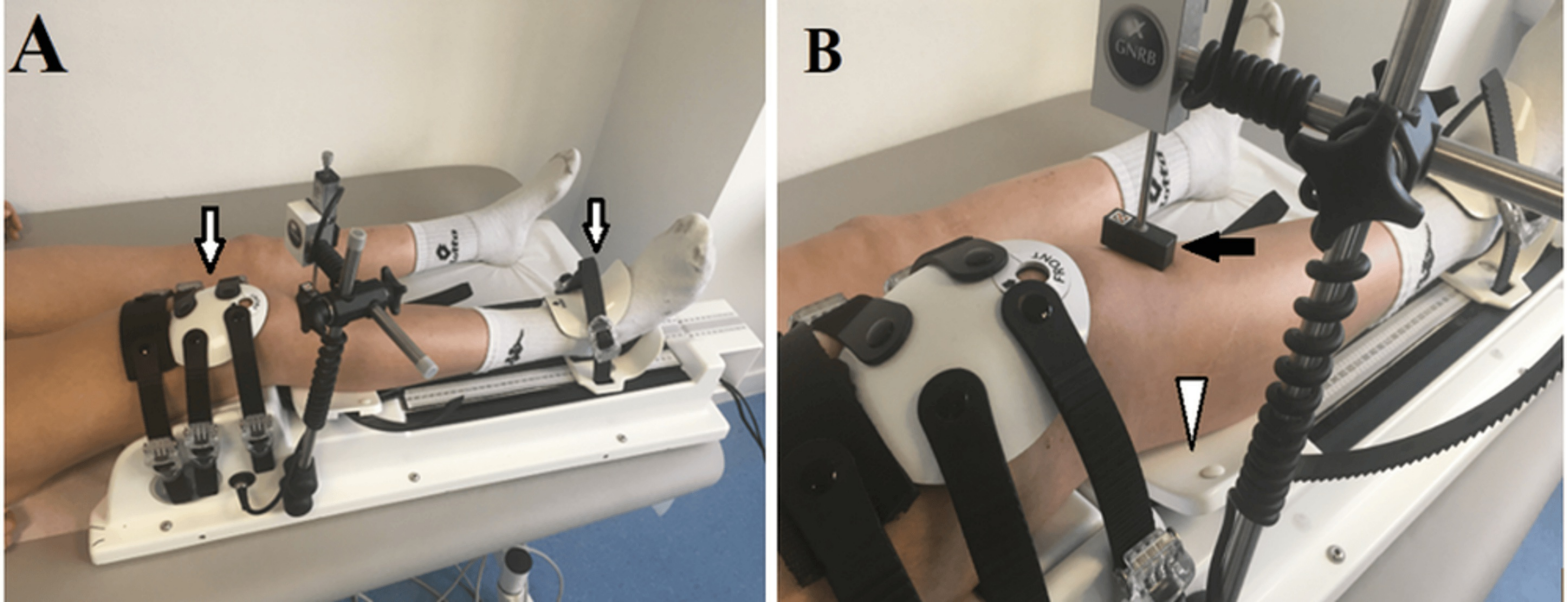
Installation of the patient in the GNRB® system* (A) The lower limb rests on the machine with two fixed points, one at the patella and the other at the ankle (white arrows); (B) The sensor is placed on the anterior tibial tuberosity two fingerbreadths from the support (black arrow) and posterior push system mimics the anterior drawer (arrowhead). *GeNouRoB, Laval, France

We performed anterior laxity measurements with the GNRB system. It was installed in a half-seated position on the examination table, with the lower limb resting on the machine with two fixed points: one at the patella and the other at the ankle. The sensor is placed on the anterior tibial tuberosity two fingerbreadths from the support. A posterior push system mimics the anterior drawer (Figure 1).

In each healthy volunteer, the first evaluator (senior evaluator) measured the right and left knees with pushing forces of 134 N and 200 N. Then, after complete deinstallation, the second evaluator (junior evaluator) performed a new measurement of the right and left knees, which was blind to the installation and the results of the first evaluator. This protocol allowed the evaluation of inter-observer reproducibility. In each volunteer, we then performed the same sequence blinded to the first one, allowing an evaluation of the inter-observer and intra-observer reproducibilities. For the evaluation of the learning curve, the junior evaluator performed a third series of measurements, with two measurements blinded to each other, to again assess its intra-observer reproducibility and progression.

Statistical methods

Inter- and intra-observer reproducibility analyses were performed by calculating Cohen’s kappa coefficient and intraclass correlation coefficient (ICC) and schematizing them according to the Bland-Altman method. Inter-observer agreement was interpreted as follows: poor agreement for values <0.45, fair to good for values between 0.45 and 0.75, and excellent for values >0.75. Statistical analysis was performed using MedCalcStatistical Software version 15.2 (2015; MedCalc Software Ltd, Ostend, Belgium). P<0.05 was considered significant.

## Results

The study included 20 healthy knees, with anterior knee laxity measurements performed using the GNRB knee arthrometer. Two different push forces (134 N and 200 N) were applied for each measurement, and intra- and inter-observer reproducibility was assessed. The characteristics of the population are shown in Table 1. 

**Table 1:** Characteristics of the population p-values were non-significant

Knee/Characteristic	Number of knees	P*
Left Knee, n	10	> 0.05
Right Knee, n	10	
Average laxity of left knees in mm for all measurements by both observers, mean ± SD	3.79 ± 1.51	> 0.05
Average laxity of right knees in mm for all measurements by both observers, mean ± SD	3.68 ± 1.25	
Average laxity of left knees at 134 N in mm for both observers, mean ± SD	2.91 ± 1.05	> 0.05
Average laxity of right knees at 134 N in mm for both observers, mean ± SD	2.81 ± 0.74	
Average laxity of left knees at 200 N in mm for both observers, mean ± SD	4.66 ± 1.40	> 0.05
Average laxity of right knees at 200 N in mm for both observers, mean ± SD	4.54 ± 1.04	
Difference in average laxity (Left - Right) at 134 N, mean ± SD	0.77 ± 0.76	> 0.05
Difference in average laxity (Left - Right) at 200 N, mean ± SD	1.07 ± 1.16	

Intra-observer reproducibility

Table 2 presents the intra-observer reproducibility for both the senior and junior examiners. The senior examiner demonstrated excellent reproducibility, with an ICC greater than 0.75 at both 134 N and 200 N. For the junior examiner, the reproducibility was fair to good, with ICC values ranging between 0.45 and 0.75 at the same force levels. However, when considering all measurements across the entire population (both 134 N and 200 N), the junior examiner also achieved excellent reproducibility (ICC > 0.75).

**Table 2:** Intra-observer reproducibility intra-observer reproducibility was excellent for the senior examiner (Examiner 1) at 134 N, 200 N, and over the whole population. Intra-observer reproducibility was fair to good for the junior operator (Examiner 2) at 134 N, 200 N. CI: confidence interval; SE: standard error; ICC: intraclass correlation coefficient

	134 N (Examiner 1)	134 N (Examiner 2)	200 N (Examiner 1)	200 N (Examiner 2)	134 N and 200 N (Examiner 1)	134 N and 200 N (Examiner 2)
ICC	0.8089	0.6460	0.8186	0.6658	0.8760	0.8099
CI	0.5758 - 0.9200	0.2938 - 0.8434	0.5658 - 0.9220	0.3202 - 0.8537	0.7775 - 0.9325	0.6685 - 0.8948
Kappa	0.788 (SE 0.077)	0.584 (SE 0.180)	0.746 (SE 0.087)	0.555 (SE 0.186)	0.854 (SE 0.042)	0.802 (SE 0.059)
CI	0.632 - 0.935	0.231 - 0.936	0.575 - 0.917	0.190 - 0.920	0.773 - 0.936	0.687 - 0.919

**Figure 2:**
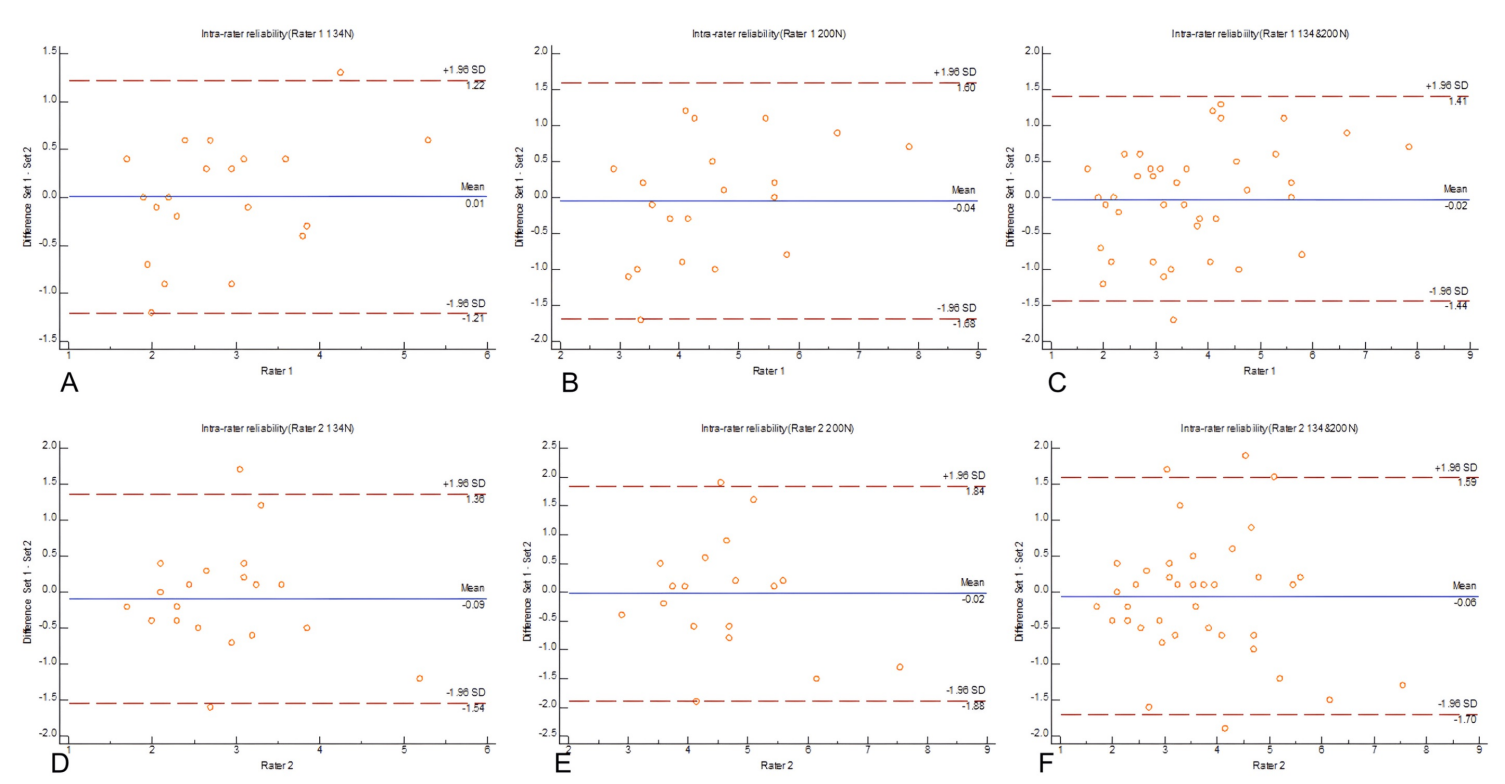
Intra-observer reproducibility between rater 1 and rater 2 using Bland-Altman method for different raters measurements force The difference between two sets of ratings (Set 1 and Set 2) is plotted against the mean rating for each rater. The solid blue line represents the mean difference, while the dashed red lines represent the limits of agreement (± 1.96 SD). (A) Intra-rater reliability for Rater 1 (134 N), showing a mean difference of 0.01 with limits of agreement (+1.22, -1.21). (B) Intra-rater reliability for Rater 1 (200 N), showing a mean difference of -0.04 with limits of agreement (+1.27, -1.65). (C) Intra-rater reliability for Rater 1 (Combined: 134 N and 200 N), showing a mean difference of -0.02 with limits of agreement (+1.47, -1.44). (D) Intra-rater reliability for Rater 2 (134 N), showing a mean difference of -0.09 with limits of agreement (+1.35, -1.54). (E) Intra-rater reliability for Rater 2 (200 N), showing a mean difference of -0.02 with limits of agreement (+1.57, -1.65). (F) Intra-rater reliability for Rater 2 (Combined: 134 N and 200 N), showing a mean difference of -0.06 with limits of agreement (+1.59, -1.70). Rater 1 = senior examiner; Rater 2 = junior examiner

The Bland−Altman plots (Figure 2) show the agreement between repeated measurements for the senior and junior examiners. For the senior examiner, the limits of agreement were narrow at both force levels, indicating minimal variability between repeated measurements. The junior examiner’s reproducibility showed greater variability, especially at the 134 N force, but improved substantially when both forces were combined.

Inter-observer reproducibility

Inter-observer reproducibility, which compares measurements between the senior and junior examiners, was also assessed. As shown in Table 3, the ICC for inter-observer reproducibility was fair to good (0.72 and 0.74 at 134 N and 200 N, respectively). However, when data from both forces were combined across the entire population, the ICC improved to 0.84, indicating excellent agreement between the two observers.

**Table 3:** Inter-observer reproducibility Inter-observer reproducibility was fair to good at 134 N and 200 N, while it was excellent for the whole population (on the first 20 cases of the junior examiner (examiner 2)). CI: confidence interval; SE: standard error; ICC: intraclass correlation coefficient

	134 N (Examiners 1 - 2)	200 N (Examiners 1 - 2)	134 N and 200 N (Examiners 1 - 2)
ICC	0.7179	0.7471	0.8402
CI	0.525 – 0.840	0.572 – 0.858	0.762 – 0.894
Kappa	0.674 (SE 0.086)	0.704 (0.074)	0.833 (SE 0.031)
CI	0.505 – 0.842	0.559 – 0.84847	0.773 – 0.893

**Figure 3:**
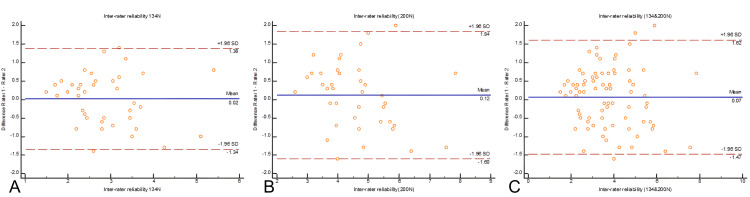
Inter-observer reproducibility between two raters for different measurements setting according to the Bland-Altman method. The difference between ratings from Rater 1 and Rater 2 is plotted against the mean of both raters' scores. The solid blue line represents the mean difference, while the dashed red lines represent the limits of agreement (± 1.96 SD). (A) Inter-rater reliability at 134 N, showing mean difference of 0.02 with limits of agreement (+1.38, -1.34). (B) Inter-rater reliability at 200 N, showing mean difference of 0.12 with limits of agreement (+1.94, -1.60). (C) Inter-rater reliability for combined measurements: 134 N and 200 N, showing mean difference of 0.07 with limits of agreement (+1.62, -1.47). Rater 1 = senior examiner; Rater 2 = junior examiner

Figure 3 depicts the Bland−Altman plot for inter-observer reproducibility. Although there is some variation between the examiners’ measurements, the overall agreement was high, particularly at the higher force of 200 N, where the limits of agreement were narrower.

The learning curve of the junior examiner

Table 4 provides data on the learning curve of the junior examiner. In the first round of measurements, the junior examiner achieved fair to good reproducibility (ICC 0.45−0.75) at both 134 N and 200 N. After completing 20 additional knee assessments, the reproducibility improved significantly to an excellent level, with ICC values exceeding 0.75 for both forces. This learning curve is also reflected in Table 5, where the agreement between the first and second rounds of the junior examiner’s measurements improved considerably after the initial 20 cases.

**Table 4:** Intra-observer reproducibility for the two examiners after training the junior operator (examiner 2) in 20 cases. Intra-observer reproducibility was excellent at 134 N, 200 N, and over the entire population for the two operators. CI: confidence interval; SE: standard error; ICC: intraclass correlation coefficient *After second round of 20 measurements by the junior examiner

	134 N (Examiner 1)	134 N (Examiner 2)*	200 N (Examiner 1)	200 N (Examiner 2)*	134 N and 200 N (Examiner 1)	134 N and 200 N (Examiner 2)*
ICC	0.8089	0.8537	0.8186	0.9185	0.8760	0.959
CI	0.576 – 0.920	0.668 – 0.939	0,566 – 0,922	0.806 – 0.893	0.777 – 0.932	0.918 – 9.976
Kappa	0.788 (SE 0.077)	0.794 (SE 0,069)	0.746 (SE 0.087)	0.880 (SE 0.044)	0.854 (SE 0.042)	0.951 (0.014)
CI	0.632 – 0.935	0.657 – 0.930	0.575 – 0.917	0.794 – 0.967	0.773 – 0.936	0.925 – 0.978

**Table 5:** The intra-observer agreement comparison between the first 20 measurements and the next 20 measurements of examiner 2 (junior examiner) shows the learning curve. The intra-observer reproducibility increased after 20 cases to excellent at 134 N and 200 N, while it was poor to good CI: confidence interval; SE: standard Eerror; ICC: intraclass correlation coefficient *second round of 20 measurements by junior examiner

	134 N, Examiner 2	134 N, Examiner 2*	200 N, Examiner 2	200 N, Examiner 2*	134 N and 200 N, Examiner 2	134 N and 200 N, Examiner 2*
ICC	0.6460	0.8537	0.6658	0.9185	0.8099	0.959
CI	0.294 – 0.843	0.668 – 0.939	0.320 – 0.8537	0.806 – 0.893	0.668 – 0.895	0.918 – 9.976
Kappa	0.584 (SE 0.180)	0.794 (SE 0.069)	0.555 (SE 0.186)	0.880 (SE 0.044)	0.802 (SE 0.059)	0.951 (0.014)
CI	0.231 – 0.936	0.657 – 0.930	0.190 – 0.920	0.794 – 0.967	0.687 – 0.919	0.925 – 0.978

## Discussion

We found good inter- and intra-observer reproducibility of the measurement of anterior laxity of the healthy knee with GNRB. The learning curve of approximately 20 cases appears sufficient to obtain excellent reproducibility. We found excellent intra-observer reproducibility for the senior assessor at 134 N and 200 N from his first series of measurements, while it was fair to good for the junior assessor. We have no explanation for these differences. The most likely explanation is a better respect for the installation and the clamping force.

We found an improvement in the results for the junior assessor in the second set of measurements. At 134 N and 200 N, intra-observer reproducibility became excellent, with reproducibility coefficients exceeding those of the senior examiner. Inter-observer reproducibility was fair to good at 134 N and 200 N (ICC, 0.7179 and 0.7471, respectively). The confidence intervals were 0.525-0.840 at 134 N and 0.572-858 at 200 N. This can be explained by a lack of statistical power. Indeed, inter-observer reproducibility was excellent over the entire population. Meanwhile, the junior examiner was on his first 20 cases when the inter-observer reproducibility was evaluated. We recall that the machine was completely out of adjustment between the two measurements. Our study also showed the absence of significant differences between the two knees in the same patient, an interesting result for studies that compare operated knees with healthy knees.

In this study, the GNRB is a reproducible means of measuring anterior knee laxity within and between observers. Initial training, good respect for the instructions for use, and a learning curve of approximately 20 cases allow any observer to use this measuring device in a reproducible way.

Colette et al. showed that GNRB had better inter- and intra-observer reproducibility than KT 1000 regardless of the examiner’s experience [5]. GNRB has also shown superiority in the diagnosis of partial ACL injury compared to Telos. Mouarbes et al. analyzed the reproducibility of GNRB measurements in 30 healthy knee subjects [6]. The findings revealed poor reproducibility under optimal comparability conditions, with low test-retest agreement for both absolute values and differentials at 134 N and 200 N forces, as evidenced by an ICC ranging from 0.210 to 0.486. Significant variations in anterior tibial translation and side-to-side difference were observed based on the applied patellar pressure, which strongly influenced the measured laxity values. Additionally, excessive patellar pressure resulted in a decrease in anterior tibial translation, potentially attributed to hamstring contraction. Klouche et al. showed that a pressure of 200 N was sufficient to diagnose complete ACL rupture [7].

Smith et al. conducted a study to evaluate the reliability of the GNRB knee arthrometer in measuring ACL stiffness and laxity [8]. Twelve healthy students underwent testing on two different occasions, and measures of anterior tibial translation and stiffness were obtained by experienced examiners. The results demonstrated good reliability, with intraclass correlation coefficients ranging from 0.72 to 0.83. The authors concluded that the GNRB device has clinical value for measuring laxity and stiffness and can assist in the design of future clinical trials.

Magdič et al. conducted a study to assess the intra-rater reliability of the GNRB knee arthrometer in measuring knee anterior laxity [9]. The study involved 97 healthy subjects, and measurements were taken by a single examiner. The results demonstrated high intra-rater reliability at forces of 134 N and 200 N, indicating consistent measurements of knee anterior laxity.

In theory, the use of laximetry measurements should be preferable to clinical testing because it provides objective measurements and does not consider the experience of the examiner. Studies have shown that clinical examination is less reproducible than laximetry [10]. In practice, it appears that the two are complementary. The diagnosis of ACL rupture is based on a combination of clinical, laximetry, and radiological evidence [11].

Although laximetry provides an objective measurement of anterior translation, it should be noted that many factors can affect its performance. Similar to the clinical examination, poor execution of the tests can lead to a poor conclusion [6].

Many methods have been described to measure sagittal laxity: the KT 1000 is the oldest and currently the most widely used device. To date, its reproducibility has been questioned. There is indeed a significant influence of the examiner on the performance of the test, the force applied manually [12]. Wiertsema et al. have shown better reproducibility of the Lachman test with the KT 1000 [13]. However, there are good results with experienced examiners, and with maximum force, a sensitivity and specificity of 93% were reached [14].

The Rolimeter® (Aircast Europa, Neubeuern, Germany) shows similar results to the KT 1000 in terms of reproducibility. Its sensitivity and specificity are close to KT 1000 [15]. However, it remains inferior to Telos, particularly for the diagnosis of preoperative rupture [16].

The Telos system is a laximetry device coupled to radiography. It has good reproducibility, but low sensitivity compared to the KT 1000 [17]. Its irradiating nature does not make it a technique of choice. In their study, Beldame et al. compared between the GNRB and Telos systems by assessing anterior knee laxity in a cohort of 157 patients with anterior ACL tears [18]. They found that the GNRB and Telos systems had similar diagnostic performance. However, they highlighted a significant advantage associated with the GNRB arthrometer, specifically its non-irradiating assessment method. This characteristic enables the potential for repeated measurements, for therapeutic and diagnostic use.

Bouguennec et al. conducted a study to assess the comparative reproducibility of the Telos and GNRB systems in measuring anterior tibial translation in normal knees [19]. Their study concluded that the GNRB device offered greater reproducibility compared to Telos, suggesting its potential as a more reliable assessment tool for quantifying anterior tibial translation. They also highlighted the limitations of Telos, including operator-dependency of measurements on radiographs, which the GNRB arthrometer aimed to mitigate. In a cadaveric study, Jenny et al. compared the GNRB arthrometer with the OrthoPilot® navigation system (B. Braun, Melsungen, Germany), assessing anterior translation in intact and transected ACLs under forces ranging from 134 N to 250 N [20]. The results indicated no significant difference between the two systems across all applied forces and ligament conditions. Furthermore, a strong correlation, good agreement, and high consistency were observed between the two measurement methods. Based on these findings, the authors recommend the utilization of the GNRB for evaluating anterior knee laxity. Despite the need for precise installation, which improves with experience, GNRB is the easiest-to-use device with the best results in inter- and intra-observer reproducibility [21].

All of these devices allow for the measurement of sagittal laxity, and considering rotational laxity could increase the sensitivity of these tests. There are other measurements of rotational laxity such as Rottometer but these are not currently used in clinical practice.

The strong point of our study was the blind measurement between the two evaluators. The change in the junior evaluator’s learning curve from mediocre to excellent results showed a certain ease in mastering the use of GNRB after only 20 measurements. Measurements were performed according to published methods [6,9].

There are some limitations to this study. First, a small number of subjects were examined and measurements were taken only on healthy knees. Second, the question of reproducibility on knees with ruptured ACLs or operated knees may be raised. Jenny et al. showed that GNRB underestimates anterior laxity by 3.7 mm in knees with ruptured ACL compared with laximetry measured intraoperatively by the navigation system [22]. Ryu et al. showed that GNRB was more effective in the diagnosis of acute ACL injury (within 10 days) compared to Lachman's test and Telos [23]. Third, current tools allow only the study of sagittal laximetry without considering rotational laximetry.

## Conclusions

The GNRB knee arthrometer demonstrated excellent inter- and intra-observer reproducibility for evaluating anterior knee laxity, especially after a brief learning curve for less experienced examiners. Our findings suggest that a learning period involving approximately 20 knee evaluations is sufficient for junior examiners to achieve reliable and reproducible results comparable to more experienced practitioners. The GNRB system offers an objective, consistent, and non-irradiating method for assessing anterior knee laxity. Although our study was limited by a small sample size of healthy knees, the results highlight the potential of the GNRB device as a valuable tool for reproducible measurements in both clinical practice and research. Future research should investigate the inter/intra observer reliability and learning curve of the GNRB device for diagnosing ACL ruptures and assessing residual laxity post-ACL reconstruction
